# Using artificial intelligence to promote equitable care for inpatients with language barriers and complex medical needs: clinical stakeholder perspectives

**DOI:** 10.1093/jamia/ocad224

**Published:** 2023-12-14

**Authors:** Amelia K Barwise, Susan Curtis, Daniel A Diedrich, Brian W Pickering

**Affiliations:** Biomedical Ethics Research Program, Pulmonary and Critical Care Medicine, Mayo Clinic, Rochester, MN 55902, United States; Biomedical Ethics Research Program, Mayo Clinic, Rochester, MN 55902, United States; Department of Anesthesiology and Perioperative Medicine, Mayo Clinic, Rochester, MN 55902, United States; Department of Anesthesiology and Perioperative Medicine, Mayo Clinic, Rochester, MN 55902, United States

**Keywords:** artificial intelligence, complex care, language barrier, health equity, non-English language preferred, LEP

## Abstract

**Objectives:**

Inpatients with language barriers and complex medical needs suffer disparities in quality of care, safety, and health outcomes. Although in-person interpreters are particularly beneficial for these patients, they are underused. We plan to use machine learning predictive analytics to reliably identify patients with language barriers and complex medical needs to prioritize them for in-person interpreters.

**Materials and methods:**

This qualitative study used stakeholder engagement through semi-structured interviews to understand the perceived risks and benefits of artificial intelligence (AI) in this domain. Stakeholders included clinicians, interpreters, and personnel involved in caring for these patients or for organizing interpreters. Data were coded and analyzed using NVIVO software.

**Results:**

We completed 49 interviews. Key perceived risks included concerns about transparency, accuracy, redundancy, privacy, perceived stigmatization among patients, alert fatigue, and supply–demand issues. Key perceived benefits included increased awareness of in-person interpreters, improved standard of care and prioritization for interpreter utilization; a streamlined process for accessing interpreters, empowered clinicians, and potential to overcome clinician bias.

**Discussion:**

This is the first study that elicits stakeholder perspectives on the use of AI with the goal of improved clinical care for patients with language barriers. Perceived benefits and risks related to the use of AI in this domain, overlapped with known hazards and values of AI but some benefits were unique for addressing challenges with providing interpreter services to patients with language barriers.

**Conclusion:**

Artificial intelligence to identify and prioritize patients for interpreter services has the potential to improve standard of care and address healthcare disparities among patients with language barriers.

## Introduction

Language barriers cause enormous challenges for patients when engaging with the healthcare system.^1–5^ Patients with language barriers experience disparities in quality of care, safety, and health outcomes, including more harm secondary to medical errors in all healthcare settings than patients who speak English.^6–11^ These disparities in quality of care and health outcomes persist for patients with complex care needs including at end of life and during critical illness.^12–14^ Our previous work and a population based study by Yarnell et al demonstrated that immigrants and patients with language barriers have increased rates of healthcare utilization and suboptimal outcomes of critical illness such as longer intensive care unit (ICU) stays, a higher likelihood of dying in the ICU, increased use of aggressive interventions as well as poor symptom management.^12^^,^^13^ Additionally, these patients are less likely to adopt comfort measures only orders and do-not-resuscitate orders despite imminent death.^13^ If and when these approaches are adopted, there is a significant delay in doing so.

A framework developed by Cooper for delivering equitable healthcare supports leveraging interpreters’ skills to reduce cultural, language and literacy barriers with the goal of improving communication between patients and clinicians.^15^ Several studies demonstrate that patients who do not have access to interpreters do poorly.^16^^,^^17^ A large amount of evidence highlights the benefits of engaging interpreters to promote improved communication, clinical outcomes, and satisfaction with care among patients.^16–21^

Interpreter inclusion as part of the healthcare team is particularly helpful during complex care needs discussions and should therefore be prioritized.^22–24^ A systematic review conducted by Silva et al highlighted that patients with language barriers experienced worse quality of end of life care and goals of care discussions when professional interpreters were not used.^23^ In-person interpreters whether doing verbatim interpretation, cultural brokering or acting as health literacy guardians are also perceived as beneficial by clinicians.^25^^,^^26^

US federal mandates entitle patients with language barriers to professional interpreter services during healthcare interactions, and these services may be provided either in-person or using virtual and remote modalities.^27–35^ Although evidence supports the use of professional interpreters in all healthcare interactions for patients with language barriers, unfortunately a shortage of interpreters means it is challenging to do this in practice.^36^^,^^37^ In addition to this barrier, currently a reliance on clinicians to take the initiative to call for an interpreter is impeding the utilization of interpreters in some clinical settings even when available. The reasons for this include a lack of awareness by clinicians of the benefits and legal requirement to use interpreters, a perception that the use of interpreters will take up valuable time, concerns about cost, and systems-factors that hamper the timely and appropriate use of interpreters at the bedside.^29^^,^^38–43^ However, studies suggest that clinicians will be more likely to use trained professional interpreters when the hospital’s organizational culture supports specific systems to facilitate this process.^44^^,^^45^ Artificial intelligence (AI) to identify patients with complex care needs and language barriers deployed as part of the clinical workflow may enable an improved process.

Artificial intelligence is a potentially powerful technology that is being increasingly deployed in healthcare.^46–54^ Machine learning and predictive analytics can be leveraged for multiple purposes including diagnosis, prognostication, and prevention of medication errors.^55–58^ We plan to use machine learning predictive analytics and workflow integration to support proactive outreach by interpreter services and prioritization to reach patients with complex care needs and language barriers. We define patients with complex care needs as those with a high burden of disease, those experiencing critical or serious illness, those with a life-limiting illness and those with palliative care needs.

Despite the potential of AI, several concerns about the use of AI in healthcare settings exist.^59–62^ Stakeholder engagement is beneficial for understanding organizational readiness and for gaining insights towards sustainable implementation.^63^^,^^64^ This qualitative study uses stakeholder engagement to understand the perceived risks and benefits of using AI to identify patients with complex medical needs and language barriers to prioritize them for in-person interpreter use.

## Methods

We conducted a qualitative study using semi-structured interviews of diverse healthcare team professionals caring for hospitalized patients with complex care needs and language barriers. The study was conducted at Mayo Clinic Rochester, a large quaternary care academic medical center with 2000 beds and 200 ICU beds. The hospital has a substantial proportion of patients with language barriers hospitalized for management of complex care issues.

The use of interpreter services is at the discretion of the healthcare team. In-person interpreters are available for several languages. In-person denotes an interpreter who is physically present at the bedside. However, if a healthcare team member prefers, or an in-person interpreter is not available to be physically present, remote video interpretation is commonly utilized using a tablet. These requests are frequently served by our institutional interpreters remotely, and if not possible using outside vendors. Remote telephone interpretation is rarely used.

Informed oral consent was obtained from participants prior to interview. Mayo Clinic Institutional Review Board approved this research (IRB 22-002974).

### Participants and recruitment

Participants were recruited via purposeful sampling^65^ with an emphasis on both variation (interdisciplinary role) and similarity (role tasks).^66^ We included ICU and hospitalist physicians, nurses, advanced practice providers, multidisciplinary therapists as well as interpreters, interpreter coordinators and health unit coordinators (all older than 18 years old). These stakeholders were selected as they either care for patients with complex medical needs and language barriers or they are integral to the process for engaging with interpreter services. We used divisional and departmental meetings to seek participants as well as word of mouth. We continued to enroll participants until data saturation was achieved within each role type.^67^^,^^68^ We experienced no difficulties with recruitment.

### Data collection and analysis

An interview guide asking a series of open-ended questions was developed by the multidisciplinary team based on literature review, expert opinion, previously conducted work in AI and language barriers, and clinical experience.^25^^,^^26^^,^^58^^,^^69–71^ We (SC) conducted one-on-one semi-structured interviews in-person and on the phone lasting approximately 30 min each between November 2022 and April 2023.^72^ Participants heard a brief description of the proposed AI tool under development to use electronic health record data to identify complex care patients located in ICU or on the floors with language barriers. Participants were then asked about how they view use of this tool to prioritize these patients for in-person interpreter services and potential benefits and risks of implementation. Interviews were audio-recorded and externally transcribed by a professional transcription firm, Landmark Associates. The transcripts were subsequently de-identified. The interview questions are included in the appendix interview guide.

Principles of grounded theory were used to analyze the transcripts with open, axial, and selective coding using the software NVivo Version 12 (QSR Intl, Burlington, MA).^73^ A codebook was developed using inductive and deductive analysis methods. The codebook was then iteratively refined using six transcripts that were deliberately sampled to include diverse participant roles. These were coded independently by two coders who met to discuss how well the codebook reflected the data and mutually agree on codebook modifications. All transcripts were then coded independently and in duplicate (AKB, SC) and coders met to reach consensus.^74^ The ongoing process of coding led to refinement of the codebook and the addition of some new codes and subcodes as well as clearer definitions of existing codes.^75^ Following completion the investigators met to develop overarching themes and select representative statements.

## Results

We interviewed 49 participants. Demographic characteristics are outlined in Table 1.

**Table 1.** Characteristics of participants.

Characteristics	LS personnel^a^ *n *=* *15	MD *n *=* *12	RN *n *=* *11	APP *n *=* *5	Other^b^ *n *=* *6
Female sex, *n* (%)	7 (43)	7 (58)	8 (72)	3 (60)	6 (100)
Age, yr, range	32-64	32-56	22-50	32-42	34-69
Race					
White	5	6	10	4	5
Black	5	1			1
Asian	0	5	1		
Choose not to answer	5				
Native American				1	
Ethnicity					
Choose not to answer	4				
Non-Hispanic	6	12	11	5	6
Hispanic	5				
Born in US=yes	6 (40)	5 (41)	11 (100)	4 (80)	4 (66)
Years experience^c^	Ave 12.3 Range 2-25	Ave 11.2 Range 5-40	Ave 6.2 Range 0-17	Ave 6.75 Range 1-14	Ave 14 Range 3-33
Languages interpreted					
Spanish	6 (40)				
Somali	4 (26)				
Arabic	1 (06)				
Mandarin	1 (06)				
Romanian	1 (06)				

a Language services personnel: interpreters and coordinators. Some coordinators speak English only.

b Other: Health Unit Coordinators, Occupational therapist, and Physical therapist.

c Years experience: working in ICU (MD, APP, RN, Other) or years interpreting (language services personnel).

The results are categorized as two major themes: perceived risks and concerns and perceived benefits. These themes each have several sub-themes. Tables 2 and 3 contain representative quotes of the major themes and sub-themes.

**Table 2.** Perceived risks and concerns themes and representative quotes.

Theme	Quote
i. Transparency about use of AI	Quote 1 “If I’m thinking as a patient, I’m thinking that you’re going to be input into this software machine, and it’s going to decide if you’re a priority or not. That’s kind of scary…” Interpreter 03
ii. Accuracy of AI	Quote 2 “Taking away that contact or the context of somebody who may need it, and we don’t identify them as one. There might be some missed opportunities potentially.” Physician 04 Quote 3 “… but how do you know who’s the priority? What’s the base? What do you base it on?” Interpreter 03
iii. Redundancy of AI in this context	Quote 4 “I think we get those alerts raised with the “needs interpreter” flag on their charts. That information is readily available for their team. They just have to know where to look, and actually look at it. …” Physician 01 Quote 5 “I feel my assessment on whether a patient qualifies for an interpreter is more than … is better than some algorithms” RN 11 Quote 6 “for my style of practice I don’t need it, but once again I would respect that people may have a different perspectives and different needs.” Physician 14
iv. Overreliance on AI	Quote 7 “I think about one of the tools that I know is the ECG AI. I think it even has a comment or something on there that says, “this is a tool … I would say, that (this AI) … this is just a tool” Physician 04
v. Privacy and Confidentiality	Quote 8 “I just don’t know exactly what is being done with that information.” RN 09
vi. Perceived Stigmatization	Quote 9 “what’s the process … because my only question is whether that in some way might mark or stigmatize a patient … sometimes people get upset if we call an interpreter … because some people don’t want an interpreter to be privy to their personal details.” Physician 07
vii. Implementation Issues/Alert Fatigue	Quote 10 “Where it’s put into practice … I don’t know … I feel we have to try to eventually know how to use it, how best to use it.” Interpreter 03 Quote 11 “the risk is pretty minimal, but it’s just over-alerting I guess.” Physician 17
viii. Supply and Demand	Quote 12 “I anticipate there will be staffing issues … how do we man that … with enough interpreters in house?” Physician 17 Quote 13 “we should be a bit more astute and sophisticated about this rather than just saying, “Oh, yeah, I need …, a Spanish interpreter, or an Arabic interpreter” because the dialects make a lot of difference, and especially the older generations, I worry that we may not getting all the nuances. Secondly, we should just have a lot more availability.” Physician 03

**Table 3.** Perceived benefits themes and representative quotes.

Theme	Quotes
i. Increase awareness of in-person interpreters	Quote 1 “I think people just default to the iPads not realizing we do have interpreters in house that can come” RN 02 Quote 2 “I have to admit that would be really cool if we got an alert—‘cause I know a lot of floors just think, “Oh. We’ll just use the iPad.” Coordinator 03
ii. Improved prioritization and resource allocation for patients	Quote 3 “AI might actually help us on the flipside, (of) saying these people probably would benefit more or need a prioritization. I think it’s good to look at it from different perspectives.” Physician 04 Quote 4 “by proactively finding these patients and providing these resources earlier on, I believe would be beneficial, not only in the care that we provide as a hospital but also the way that the patient interprets that care that they are receiving.” RN 04 Quote 5 “I think it’s great to know who does need [interpreter services], so then we can—‘cause a lot of its lost in transit. I know the hospital’s a busy place.” Coordinator 02
iii. Empower and support bedside team	Quote 6 “That decision is really one that is led by the bedside team, and so potentially you could have a situation where you have the room nurse empowered to call for an in-person interpreter without needing to go through the team first.” Physician 09 Quote 7 “I think a prompt would be helpful because sometimes, as the bedside team, I can get wrapped up in a busy morning and not always anticipate that (a patient needs an interpreter). Maybe I realize, “Oh, we’re gonna have a goal of care,” but not always have the mental capacity even to put that into the forefront of my mind. That’s not always my biggest priority. Sometimes there’s medical things that take precedent, but maybe even having that reminder could be helpful.” RN 05
iv. Enhance Interpreter Preparation and Input	Quote 8 “language services is a prime example of a support for the bedside staff. Something that can make their job better and easier for us to access (it) is what I’m excited for AI to be used for.” RN 12 Quote 9 “I think alerting the interpreter would be the biggest win … if they are prepared for that conversation and getting alerted that yes, we need them, they are going to be more prepared.” Physician 17
v. Streamline process and increase efficiency	Quote 10 “I think that it’s always good to have an AI algorithm to reduce the workload of healthcare team members and make you more efficient. Algorithms are very important to avoid redundancies” Physician 15 Quote 11 “I think what you guys are doing [study] I think is maybe the answer to the entire problem if our office [language services] knows that (there is) a patient in need- then they can call and schedule morning rounds. I think … it’d be a great tool.” Interpreter 01
vi. Improve standard of care	Quote 12 “Maybe we are missing important patients at risk for this. Maybe that’s something that we’ll be able to learn. Maybe post intervention, maybe the standard would be that we’re having more in person interpreters, versus using the iPad …” Physician 15 Quote 13 “I think most of it is good when we get interpreter in there in person versus video or the phone. I think in person we’re gonna be more beneficial to the patient, to the team, and to the interpreter as well.” Interpreter 07
vii. Overcome clinician bias	Quote 14 “I think we do (use in-person interpreters) with certain people, but I think there’s definitely a chunk of people that aren’t (getting in-person interpreters). I feel like sometimes it’s not even a thought. It’s kind of an afterthought to get an in-person interpreter.” Coordinator 03 Quote 15 “I don't think there are going to be risks and concerns. If anything, it would help us overcome our implicit biases where because of their limited English proficiency, they're—I hate to say it this way, but sort of sidelined and not communicated to. The ability to identify them at least will make us consciously do that. I don’t know that it’s risky to do[study]. It’s risky not to do it. That’s what I think.” Physician 11

### Perceived risks and concerns (see Table 2 for themes and representative quotes)

We asked participants for responses to the use of AI for identifying and prioritizing patients with language barriers and complex care needs and then probed to uncover their concerns. When asked specifically about the use of AI, our participants’ responses echo discussions of ethical use of AI in healthcare overall.^76^ Participants’ expressions of concern and identification of risks relating to the use of AI for patients with language barriers and complex care needs were less frequently mentioned by participants and less strongly articulated in contrast to their descriptions of benefits.

#### Themes related to risk included

##### (i) Transparency about use of AI

Participants commented about transparency of use, noting that patients might like to be aware that an algorithm was involved in prioritizing them for interpreter services. Participants also noted that patients might wonder why they were selected and what specific criteria is stored and used for this purpose (Quote 1).

##### (ii) Accuracy of AI

Other participants expressed doubt about the accuracy of the algorithm itself to identify and prioritize the patients most in need of interpreter services (Quote 2, Quote 3).

##### (iii) Redundancy of AI in this context

Some clinicians objected to the need for AI to be used as we proposed, pointing out that the information in the electronic medical record (EMR) and observation of the patient’s ability to communicate is sufficient to ascertain if they warrant interpreter services (Quotes 4 and 5). These providers viewed the AI as superfluous and proclaimed their use of interpreters to be appropriate (Quote 6).

##### (iv) Overreliance on AI

Participants cautioned against over reliance on AI, stressing its use as a tool rather than a replacement for decision making (Quote 7).

##### (v) Privacy and confidentiality

Participants expressed few concerns related to loss of privacy or confidentiality for patients but several raised concerns over potential unknown uses of data related to patients’ language proficiency (Quote 8).

##### (vi) Perceived stigmatization

Some participants noted that occasionally patients reject interpreter services because they feel their English language proficiency is adequate or they prefer to communicate independently with the healthcare team or using family (Quote 9). Some noted that patients may feel stigmatized by being identified as needing an interpreter and refuse services on that basis.

##### (vii) Implementation, including alert fatigue

Logistical concerns identified by participants related to how the AI would be integrated into the existing clinical workflow. Many wondered how patient prioritization would be communicated to the patient’s healthcare team (Quote 10) and if that communication itself would result in an additional burden. The most common concern among physician participants was alert fatigue (Quote 11).

##### (viii) Supply–demand issues

Some feared that the lack of sufficient in-person interpreters would result in disruption of care due to long wait times (Quote 12, Quote 13). These participants recalled situations in which they could not obtain an interpreter who spoke the same dialect as their patient, or out of hours scenarios when they perceived interpreters were not available.

### Perceived benefits (see Table 3 for themes and representative quotes)

All participants agreed that in-person interpreters improve the patient experience and are especially important during complex care decision making and were supportive about increasing the use of interpreters for patients with complex care needs “I think when teams recognize that an interpreter is going to be key to patient care, that is always a win for our patients and, I think, for our staff” Interpreter 08.

#### Themes related to benefit included

##### (i) Increase awareness of in-person interpreters

Currently, healthcare teams often rely on video interpretation using a tablet (iPad), both out of habit and immediacy of need (Quote 1). While nearly all participants noted technical issues and acknowledged lower quality of communication when using a tablet (iPad), not all were aware of the availability of in-person interpreters in the hospital setting. This is due in part to pandemic restrictions which limited use of in-person bedside interpreters. Many participants, especially interpreters and coordinators, felt that highlighting the availability of in-person interpreters in the hospital setting was a benefit for everyone (Quote 2).

##### (ii) Improve prioritization and resource allocation for patients

Participants also recognized the potential ability of AI to identify and prioritize patients (Quote 3). These participants appreciated the idea of simplifying access to a service they view as beneficial to their patients (Quote 4). Improved allocation of interpreter resources to those most in need was noted as a key benefit by participants in all categories (Quote 5).

##### (iii) Empower and support healthcare team/bedside nurses

Some participants imagined an AI-generated alert could serve as a nudge or even an imperative to take the steps necessary to ensure a patient receives interpreter services. Overall, this could potentially empower any member of the healthcare team to arrange for in-person interpreter services without hesitating to use a scarce resource and without relying on a request by a physician (Quote 6, Quote 7).

##### (iv) Enhance interpreter preparation and input

Interpreters noted that use of AI to identify patients would provide them with more timely notification and thus allow them to prepare for the interaction with the patient and family (Quote 8). This was seen as beneficial and an enhancement of their ability to effectively interpret complex discussions and increased perceived valuation of their skills (Quote 9).

##### (v) Streamline process and improve efficiency

Participants noted the potential for AI to improve the current processes for deploying interpreters throughout the hospital system (Quote 10). These participants were enthusiastic about an application of AI that solves a recognized systemic logistical issue (Quote 11).

##### (vi) Improve standard of care

Participants noted that in-person interpreters make clinicians’ jobs easier by improving the quality of communication with patients and providing context in situations where cultural approaches to healthcare decision making affect the dialog between patient, family, and clinician. In addition to those who pointed out benefits to individual patients, some participants identified benefits of the AI for the institution overall improving access to in-person interpreters as a new standard of care (Quote 12, Quote 13).

##### (vii) Overcome clinician bias

Participants pointed out that an AI tool might be fairer than the current system which relies on clinician initiative to seek an in-person interpreter. Participants noted that the AI might alert them to consider interpreter services more often and for more patients (Quote 14). A few suggested the tool might overcome implicit bias that causes some clinicians to hesitate calling interpreter services potentially due to concern about inherent challenges in scheduling (Quote 15).

## Discussion

This qualitative research study explored stakeholders’ perceived risks and benefits about using AI to identify inpatients with language barriers and complex medical needs to prioritize interpreter services. This is the first study that elicits stakeholder perspectives on the use of AI with the goal of improved clinical care for inpatients with language barriers. Participants noted both risks and benefits to the use of AI in this domain. Some of these risks and benefits overlap with already flagged hazards and values of AI but some were new and unique to the use of AI in this realm and are described below.^76–78^

Risks cited by participants related to the integration of the AI into the workflow and the potential for alert fatigue, redundancy, perceived stigmatization, supply–demand issues as well as transparency, accuracy, and privacy issues. Benefits mentioned by participants included the potential for the AI to increase awareness of in-person interpreters, improved standard of care and prioritization for interpreter utilization, a streamlined process to engage interpreters, empowered clinicians and potential to overcome clinician bias.

### Risks

Several participants were concerned about alert fatigue, secondary to notifications about patients needing interpreter services. This concern has also been shown to be common in other studies when AI is used for clinical decision support regarding medication interactions.^79–81^ Related to this were concerns about the implementation of the AI into the workflow. These views appeared to be based on prior experience with inefficient interpreter services and frustration with shortages and delays. Some participants worried that active identification of patients with complex medical needs and language barriers across the institution might cause “supply and demand issues” particularly for less commonly interpreted languages. Several studies show that AI has the potential to increase clinician workload and some participants we interviewed did convey concerns about AI creating more burden through inefficient incorporation into the clinical workflow.^82^ This exposes a tension between the promotion of AI as a means of addressing shortages in the healthcare workforce and its potential to increase clinician workload or otherwise create inefficiencies.

Some participants commented that the AI was not necessary as they felt they identified need for interpreter services appropriately. These participants likely did not appreciate the benefits of the AI score to prioritize service provision from the standpoint of institutional interpreter services, something no single human could do even if they did effectively advocate for interpreter services for their own patient. Although we recognize that health information technology including AI cannot be successful unless integrated, implemented, and accepted by the healthcare team, we do not anticipate that the likelihood of the intervention being successful will be determined by the small number of participants who thought the AI was unnecessary. However, further exploration of these perspectives is warranted during implementation.^65^

Our previous work using machine learning algorithms to encourage use of palliative care consults for patients identified as benefiting from palliative care served as a nudge to clinicians to engage with palliative care services.^58^^,^^71^^,^^83^ The proposed AI discussed here will similarly serve as a nudge to promote improved use of interpreters but if a shortage of interpreters occurs, the evaluation of medical complexity can guide interpreter services for allocation of resources.

Some participants expressed some concern about the reliability and accuracy of the AI and whether it might erroneously detect the wrong type of patients, in other words patients without complex medical needs or language barriers. These are common types of fears about AI in healthcare and other studies have also demonstrated these perceived risks about AI in other contexts.^84^ Trust in AI is a fundamental cornerstone for its successful adoption into practice.^85^ It may be related to accuracy and understandability.^78^ Notably lack of trust in AI was not articulated as a distinct concept by our participants.

As with most AI, a few participants had concerns about privacy and confidentiality however this was not a strong feature of our participants’ responses.^86^ Participants did voice some concerns about how information was handled and shared and who would review it particularly during implementation of the AI.

A few risks that participants verbalized were not uniquely related to the AI but related to identifying interpreter needs among patients generally. For example, refusal to use an interpreter when a patient might worry about their medical care being discussed in their close-knit community relate to the tenets of interpretation ethics and the dual role interpreters often retain.^87^^,^^88^ The concern about perceived stigma among patients, while potentially exacerbated with the knowledge AI had been used, is not unique to the use of AI per se and could also apply if the healthcare team requested interpreter services directly without using AI.

Overall, we found that the risks identified were similar to perceived risks of AI in other domains.^89^ However, one notable difference was that no participants voiced any negative perceptions about onus of legal responsibility if a patient was identified and interpreter services not engaged.^90^ Where the legal responsibility falls when AI is used for clinical decision support when integrated into practice is a commonly cited source of disquiet among clinicians.^91^^,^^92^

#### Perceived benefits

As well as the inherent benefits of in-person interpreters that almost all participants endorsed, the AI was considered by many as a potentially useful mechanism to remind the healthcare team that in-person interpreters were available in the institution and should be used. Anecdotal evidence as well as work we have conducted has demonstrated that the COVID-19 caused a paradigm shift in how interpreter and language services have been provided to patients over the last three years.^93^^,^^94^ While it is true that many institutions always relied exclusively on remote phone and video interpretation, institutions that had integrated in-person interpreters into the clinical practice, almost completely switched to remote interpretation.^93^^,^^94^ With high staff turnover during the pandemic years many staff have only ever used remote video interpretation, and some are not even aware of the presence of in-person interpreters in the institution. Furthermore, even among those who are aware of these services and previously used them and vouch for the importance and benefits of in-person interpreters, the usability and immediacy of the video has made it challenging to change clinician behavior to re-adopt in-person interpreters after pandemic safety measures have been discontinued.

Although the issue of bias woven into and perpetuated within AI-generated algorithms is often cited as a potential harm of AI, participants in our study noted the potential for this type of AI to overcome clinician bias that might have prevented them from using interpreter services.^95^^,^^96^ There are several barriers to the use of in-person interpreters including the challenges with easily arranging and coordinating convenient times.^26^ This deters many clinicians from using in-person interpreters and sometimes causes delays in communication, avoidance of communication or suboptimal communication without in-person interpreters. The finding that the use of AI in the manner we propose could overcome bias and therefore increase communication and potentially reduce disparities in communication and care is encouraging.

Several participants noted that in their own experience, interpreters may have been deployed to a lower priority setting while another patient in a more critical setting is thus deprived of their services. These participants recognize the value of AI for prioritizing in-need patients across the whole institution, a task not possible for the human interpreter coordinators. AI has been used to improve prioritization in other domains such as radiology and in the ER.^97–99^ In this way stakeholders supported the benefits of the AI providing some kind of objectivity of patient need.

Nurses commented that AI could support their work by acting as a reminder to engage interpreter services and some participants envisioned bedside nurses empowered by the AI (when integrated into a streamlined process) as it could serve as a mechanism that could galvanize them to engage interpreter services without the need to seek input from the healthcare team. Successful integration of AI into fields such as oncology can enhance clinicians ability to provide personalized, effective and evidence-based care.^100^ Work by Li et al has indicated that AI has the potential to empower clinicians to collaborate and communicate more effectively when identifying patients with clinical deterioration.^101^ In our study integration of the AI into the workflow was perceived by most participants to be beneficial and likely to streamline the process of accessing and providing interpreter services and increase efficiency.^101^

Many participants, especially physicians, stated that the interpreters helped them understand the cultural considerations of patients and families, a key benefit not replicated with remote video interpretation. Our previous qualitative work suggests that clinicians value in-person interpreters for this reason and perceive that poor communication and a failure by healthcare teams to understand and acknowledge sociocultural differences for patients and families with language barriers are contributing factors to suboptimal decision making, poor quality care, and disparities in outcomes.^69^ The importance of in-person interpreters for discussions involving patients with complex medical needs cannot be overstated. National organizations are increasingly recognizing the importance of good communication for high quality decision making and the role of language and cultural values for achieving care that aligns with cultural and spiritual values.^102^^,^^103^

Wang’s work describes autonomy, beneficence, explainability, justice, and non-maleficence as the five key signals of AI responsibility for healthcare.^104^ Explainability is a term that denotes that AI can be understood.^105^ The participants in our study alluded to these concepts in only a very general way. Similarly related terms such as transparency were not widely raised as concepts by our participants.^106^^,^^107^

We examined whether responses differed by role. From a risks and concerns perspective, for example most bedside nurses were very satisfied with video interpretation and expressed concerns about delays during coordination of in-person interpreters. Some even worried that access to video interpretation would be removed from them. In contrast, most physicians reiterated the value of in-person interpreters, but a few physicians voiced concerns about electronic medical record related alerts, potential stigmatization, and redundancy. Interpreters and coordinators were most likely to voice concerns about using AI for tasks currently done by a human.

In terms of benefits, all roles recognized the value of in-person interpreters and that AI could increase awareness of the potential availability and worth of in-person interpreters. Since bedside nurses are often charged with arranging in-person interpreters for the healthcare team, they were more likely to support the idea of using AI to streamline the process although some physicians and interpreters noted this benefit too. Interpreters believed using AI to help prompt healthcare teams to use an in-person interpreter would help coordinators better anticipate the needs and demand around the institution, potentially giving interpreters time to prepare for in-person encounters.

Strengths of the study include the following. We deployed robust qualitative methods, with two coders coding each transcript independently and in duplicate. We reached consensus at weekly meetings. Moreover, we were able to triangulate our data by interviewing diverse groups (physicians, bedside nurses, advanced practice providers and those working in interpreter services).^108^ These measures enhance the scientific rigor and validity and ensured the trustworthiness of our findings, strengthening the relevance of our discoveries.

This study also has several limitations. This was a single center study therefore the findings may not be generalizable to other institutions. Several participants had limited knowledge about AI in general and its potential risks and benefits of AI, with some misunderstanding the function of the AI in this work. A few participants misunderstood the AI algorithm and believed it was using electronic medical data parameters to determine English language proficiency rather than medical complexity.

Some participants, especially bedside nurses did not have experience with in-person interpreters so could not provide much perspective on the benefits or optimal use of in-person interpreters and were satisfied with remote video interpreters. Future work to gather more focused clinician perceptions might consider the use of patient and clinician scenario vignettes, as well as use of a graphic user interface in which the AI identified patients with language barriers are prioritized using a complexity score and a proposed workflow for integrating the AI into practice is clearly presented for feedback.

As well as understanding the perceived risks and benefits of the use of AI in this domain we acknowledge that in order for AI to benefit patients, it must be integrated effectively into clinical practice workflows.^51^^,^^56^^,^^109^^,^^110^ This work does not include specific issues about implementation unlike some other work.^111^ Furthermore, like any clinical intervention, a predictive model, its deployment, and its implementation and impact should be subject to robust evaluation beyond simply assessing the predictive accuracy and reliability.^112^^,^^113^

In addition, we should note that ascertaining the perspectives of patients and community members about the use of AI in healthcare generally as well as for specific tasks is important.^62^ We acknowledge that not including patient voices in this work is a limitation and an area for future study. Although we did not seek patient perspectives in this study, we did engage with our institution’s Health Data and Technology Community Advisory Board (CAB). The CAB meets monthly and invites investigators using AI to present their research. When we discussed this research program, why we believed it was important as well as the perceived risks and benefits we received positive feedback from the community members at the CAB overall. We plan to present again in the future.

The stepped-wedge cluster randomized trial that will evaluate the efficacy of the AI tool to prioritize patients with language barriers and complex medical needs for interpreter services is currently underway. However, unique potential benefits and concerns of the use of AI in this realm are outlined in a textbox below and are based on those noted by participants.

## Conclusion

We conducted a qualitative study using semi-structured interviews of diverse clinical stakeholders who highlighted potential benefits related to the use of AI when caring for patients with language barriers. This specific application of AI may remediate gaps in clinician knowledge and training about when to use in-person interpreters; may effectively support prioritization for interpreters if shortages; can highlight an important human resource to optimize face to face communication and may support best practice to address health disparities, quality, and safety. Risks and concerns must be continually evaluated but these were voiced less frequently and less vociferously than the potential benefits noted by participants.

Textbox: Unique perceived potential benefits and concerns of use of AI to promote equitable care for patients with language barriers.

Benefits

- Alerts remediate gap in healthcare team/clinician training on use of in-person interpreter at the bedside.
- Effectively supports prioritization of interpreters across large institution if shortages occur.
- Uses technology to highlight a human resource to optimize face to face communication for patients with language barriers.
- Promotes best practice to improve quality and safely to address health disparities among patients with language barriers.

Concerns

- Some clinicians already ascertain information about when to use an interpreter without use of AI.
- If identify need for interpreters, AI may highlight Supply–Demand challenges with in-person interpreter availability.
- Using AI specifically to identify patients needing an interpreter may cause patients to feel stigmatized.

## Supplementary Material

ocad224_Supplementary_Data

## Data Availability

All data relevant to the analysis are incorporated into the article.
