# Supplementary material for: Using artificial intelligence to promote equitable care for inpatients with language barriers and complex medical needs: clinical stakeholder perspectives
Source: J Am Med Inform Assoc. 2023 Dec 14;31(3):611–21. doi: 10.1093/jamia/ocad224 (PMC10873784; doi:10.1093/jamia/ocad224)
Supplement: ocad224_Supplementary_Data [file ocad224_supplementary_data.docx]

*This interview study is gathering perspectives about a future study we are planning that will use an AI tool that will utilize EHR data to identify patients who need an interpreter (preferred language not English) and have a complex illness. The AI tool will calculate a score based on the patient’s status and notify language services of their location. Language services will then contact the patient’s care team and ask if and when they would like an interpreter at the bedside. We want to know what you think about use of an AI tool for this purpose and also about the logistics of getting interpreters to the bedside when they are most needed. We are talking to physicians, nurses, interpreters, coordinators, and Health Unit Coordinators because all of the these have a role in getting interpreters to the bedside when needed.*

|  | *Topic question* | *probes* |
| --- | --- | --- |
| 1 | *When you want to get an interpreter for a patient what is your usual process?* | *What do you know about how the process for requesting interpreters works?*  *Do you see any concerns with the process used to request interpreters?*  *Have you had any issues with the process or things you’ve noticed could have been avoided/done better?*  *Do you feel you are being called to patients that most need you?*  *Do you feel you are interpreting for the patients that most need you?* |
| 2 | *What works in this approach?* | *What is currently working well?*  *How does the coordinator role help or hinder this process (interpreter)?*  *What is your experience linking up with the provider teams?* |
| 3 | *What could be improved?* | *What could be improved in your opinion?*  *Probes on: Avoiding Delays, Avoiding Rescheduling, Non availability of clinical team, No background information, suggestions for Efficiency?* |
| 4 | *What would be an ideal way to help facilitate interpreters/clinician/patients and families with LEP to meet in a timely manner?* | *Do you have suggestions for how we could link interpreter services to patients who need them?*  *Probes on: Alerts, proactive reminders to team, Drop in to interpret, Text page, Secure chat, Chart flag, Room flag, Phone call, Create Epic order* |
| 5 | *Do you think pro-actively identifying patients and alerting their healthcare teams that they might benefit from an interpreter is a good idea and helpful?* | *Why or why not?* |
| 6 | *If you were designing a tool for this purpose, which patients would you prioritize?* | *Which patients do you think have the highest need for an in-person interpreter? What considerations do you make when prioritizing in situations where there are competing needs or requests?* |
| 7 | *If you were implementing a tool like this, how would you go about getting interpreters*  *to the bedside?* | *From your vantage point, can you suggest ways that we can improve the process for getting in person interpreters to the bedside?* |
| 8 | *Do you have any concerns about an AI tool for this purpose? What are they? Do you see any risks?* | *If no concerns noted: The tool uses a score based on the patient’s medical status to prioritize them. Can you think of any risks of using AI to prioritize patients in this way?*  *Creat more demand than group could handle?*  *Clinicians feeling less in control?* |
| 9 | *Do you have any other thoughts about linking patients and families with their healthcare team to interpreters? What might we be missing? What would you like the research team to know?* | *Now that we have been talking about this project for a while, has anything else come up in your mind you could share with me?* |
